# Integrated dataset of satellite-derived water quality parameters and bird populations in five Ramsar Wetlands of India using Sentinel‑2 Imagery and in-situ observations

**DOI:** 10.3897/BDJ.14.e177883

**Published:** 2026-04-22

**Authors:** Kavya Raj, M U Sreeja, Abin Oommen Philip

**Affiliations:** 1 CR’EIA Crime Research and Edge Intelligent Agents lab, Indian Institute of Information Technology Kottayam, Kottayam, India CR’EIA Crime Research and Edge Intelligent Agents lab, Indian Institute of Information Technology Kottayam Kottayam India

**Keywords:** wetland ecosystem monitoring, biodiversity assessment, remote sensing analysis, geospatial integration, environmental health indicators

## Abstract

**Background:**

Water birds serve as particularly valuable indicators of wetland health since wetlands provide critical feeding, foraging, nesting, roosting and breeding habitats for these species. Monitoring waterbird populations over time is, therefore, essential for assessing wetland ecosystem health. To achieve this, certain wetlands are designated as Ramsar sites by identifying those with high conservation importance and implementing conservation measures.

**New information:**

To address the critical need for integrated monitoring of wetland water quality and biodiversity, where bird populations serve as key bioindicators of environmental health and ecosystem integrity, the proposed dataset provides detailed, multi-year records of water quality parameters and bird populations across five Ramsar wetlands in India: Ashtamudi, Vembanad and Sasthamkotta in Kerala; Harike Wetland in Punjab; and Point Calimere in Tamil Nadu. The data span from 2016 to 2024 for the Kerala sites and from 2018 to 2024 for Harike Wetland and Point Calimere. Using Sentinel-2 satellite imagery combined with site-specific shapefiles, surface reflectance data for each wetland are processed and analysed. For the three Kerala wetlands, seven key water quality parameters: total suspended solids (TSS), turbidity, conductivity, pH, ammoniacal nitrogen (NH₃-N), biochemical oxygen demand (BOD) and dissolved oxygen (DO) were estimated by calibrating models with in-situ measurements. In Harike Wetland and Point Calimere, the focus was on chlorophyll-a (Chl_a), cyanobacteria cell concentration (Cya), turbidity, coloured dissolved organic matter (CDOM)and dissolved organic carbon (DOC). Bird population data, centred on both migratory and resident water birds, are sourced from multiple resources and organisations, including eBird:, World Wide Fund for Nature – Kerala (WWF), Bird Count India, Kottayam Nature Society, Cochin Natural History Society (CNHS), Pathanamthitta Birders and Kollam Birding Battalion, to provide comprehensive insight into wetland biodiversity. By integrating geospatial, spectral, field and ecological information in correlation with bird population data, this dataset enables long-term tracking of wetland health and provides a foundation for bird occupancy prediction for researchers working in remote sensing, ecology and conservation. With the regression equation formulated from the collected dataset, the system could achieve a promising R² value, which indicates that this model can be further applied to these wetlands.

## Introduction

Wetland ecosystems have a strong link between water quality and bird biodiversity, as the condition of water directly affects bird populations. Although wetlands cover only 6% of the Earth’s land surface, they support a large variety of species, with waterbirds acting as important indicators of ecosystem health (Davidson and Finlayson 2018). Factors like water clarity, nutrient levels and oxygen content play a major role in shaping which bird species are present, how many there are and how diverse the bird community is (Fraixedas et al. 2014). Wetlands also provide services worth over $47 trillion each year and support more than 40% of bird species through food chains that depend on clean water (Costanza et al. 2014, Gardner et al. 2015). However, wetlands are disappearing quickly; about 64–71% have been lost since 1900, making them the fastest vanishing ecosystem type (Davidson 2014, Hu et al. 2017). This loss is mainly caused by farming, urban growth, pollution and climate change, which have led to waterbird population declines of up to 81% (Junk et al. 2012, Guillemain and Hearn 2017). Current methods for monitoring wetlands often look at water quality and bird life separately, which creates gaps in understanding and makes it harder to manage these ecosystems in a connected and effective way (Kingsford et al. 2016). Recent advancements in satellite remote sensing have significantly improved wetland monitoring, with waterbirds recognised as reliable indicators of ecosystem health due to their sensitivity to environmental changes and role in aquatic food webs (Howe et al. 2007, Gorelick et al. 2017) The multispectral capabilities of modern satellites enable detection of subtle environmental variations that directly influence avian habitat quality and distribution patterns. Sentinel-2 satellites offer high-resolution imagery (10–20 m spatial resolution) with a 5-day revisit frequency that enables tracking of key water quality parameters, such as turbidity, chlorophyll-a and suspended sediments, with satellite-derived data showing strong alignment with field measurements (Dörnhöfer and Oppelt 2016, Pahlevan et al. 2020). The Sentinel-2 multispectral images provide spectral information across visible, near-infrared and shortwave infrared regions, enabling water quality retrievals through atmospheric correction and bio-optical models. Integrating remote sensing with bird surveys supports comprehensive ecosystem monitoring using machine-learning algorithms. (Peterson et al. 2018, Yang et al. 2022). India hosts 75 Ramsar wetlands spanning over 1.3 million hectares, offering diverse habitats across climatic zones and supporting more than 400 waterbird species, including globally threatened taxa (Bassi et al. 2014). Despite their ecological importance, integrated datasets linking water quality and bird populations remain limited. To address this gap, we present a multi-site dataset combining satellite-derived water quality metrics with bird population records from five Ramsar wetlands across India. This is the first coordinated effort to align water quality and avian data at this scale in the Indian context, enabling comparative analysis across wetland types, seasonal patterns and long-term trends. The dataset includes Sentinel-2-derived water quality parameters and bird population data from eBird, CNHS, Bird Count India and regional ornithological organisations, serving as a valuable resource for wetland researchers, conservationists and policy-makers towards evidence-based ecosystem management. The integrated dataset of water quality parameters and bird counts derived from the same wetland sites also provides a foundation for future species occupancy prediction studies. The developed framework is adaptable beyond water quality applications to other environmental monitoring domains, such as soil quality assessment, air pollution studies and forest health evaluation. This research supports environmental managers and researchers with empirical data for wetland management, pollution control and biodiversity conservation, enabling broader scientific applications and conservation planning.

### Data description

A multi-year dataset of water quality parameters derived from Sentinel-2 multispectral satellite imagery has been compiled for five Ramsar wetlands in India: Vembanad, Sasthamkotta and Ashtamudi in Kerala; Harike Wetland in Punjab; and Point Calimere in Tamil Nadu. Site-specific boundaries were defined using QGIS shapefiles and satellite images spanning 2016 to 2024 for Kerala wetlands and 2018 to 2024 for the other sites were downloaded from the Copernicus Open Access Hub. The imagery, organised by wetland, year and month, includes thirteen spectral bands that were resampled to a consistent ten-metre spatial resolution. Multiple linear regression models, developed from in-situ measurements and corresponding spectral reflectance, were applied to the full dataset to estimate key water quality parameters, including total suspended solids, turbidity, conductivity, pH, ammoniacal nitrogen, biochemical oxygen demand and dissolved oxygen (Beck et al. 2019, Li et al. 2020, Sent et al. 2021, Mamun et al. 2024) for Kerala wetlands; and chlorophyll-a, cyanobacteria concentration, turbidity, coloured dissolved organic matter and dissolved organic carbon for Harike and Point Calimere. The dataset is stored in tsv format, organised by location and date, enabling detailed spatial and temporal analysis of water quality across these ecologically important wetlands. Bird population data for all five wetlands were compiled from multiple reliable sources, including eBird, Kottayam Nature Society, Cochin Natural History Society (CNHS), Bird Count India and other regional ornithological organisations, covering both migratory and resident waterbird species for comprehensive avian biodiversity assessment.

## General description

### Purpose

- Provides a validated multi-year dataset integrating satellite-derived water quality parameters with in-situ measurements for five Ramsar wetlands in India, enabling assessment of aquatic ecosystem health over time;
- Offers processed Sentinel-2 imagery resampled to consistent spatial resolution for reliable estimation of seven important water quality variables through regression modelling;
- Includes bird population data for migratory and resident waterbirds from the eBird database, supporting studies on biodiversity responses to water quality changes;
- Provides researchers and environmental managers with empirical data for wetland health analysis, management, pollution control and bird occupancy prediction modelling, promoting their reuse for scientific and conservation purposes.

## Sampling methods

### Sampling description

**Figure 1.**
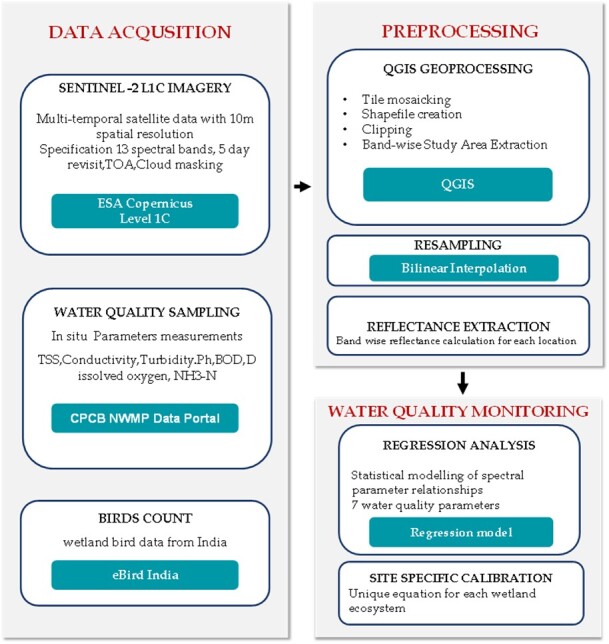
Methodological framework for multi-source wetland data integration.

The study employed a systematic approach to create an integrated dataset for wetland characterisation. Fig. 1 illustrates the methodological framework comprising three phases: (i) Data acquisition of Sentinel-2 imagery, water quality parameters and bird count data; (ii) Preprocessing including geoprocessing, resamplinga nd reflectance extraction; and (iii) Statistical analysis involving regression modelling and site-specific calibration.

**Figure 2.**
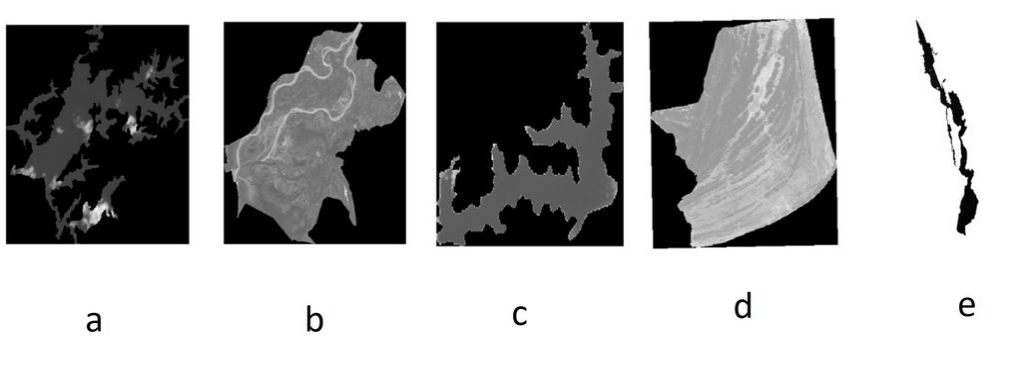
Clipped Sentinel-2 raster images of the five Ramsar wetlands.

**Figure 3.**
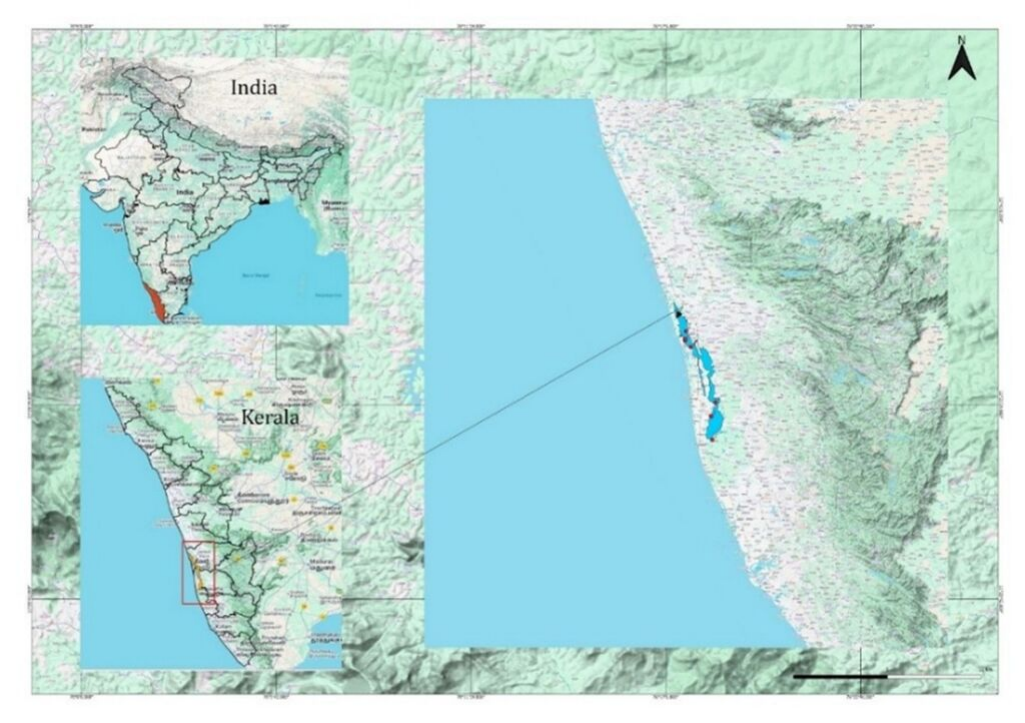
Vembanad Lake and the sampling points.

The study focuses on five Ramsar wetlands in India: Vembanad, Sasthamkotta and Ashtamudi in Kerala; Harike Wetland in Punjab; and Point Calimere in Tamil Nadu. Sentinel-2 images with a cloud cover threshold of ≤ 11% were downloaded from the Copernicus Open Access Hub. Atmospheric correction was performed using the DOS1 method via the Semi-Automatic Classification Plugin (SCP) in QGIS. For each wetland, individual shapefiles were created using QGIS to accurately define their spatial boundaries. These shapefiles were then used to clip Sentinel-2 satellite images, producing wetland-specific raster datasets (Toming et al. 2016). Surface reflectance was subsequently extracted using the standard ESA quantification formula:Surface Reflectance = DN/10000. Fig. 2 presents the clipped Sentinel-2 satellite imagery for the five study wetlands: (a) Ashtamudi, (b) Harike Wetland, (c) Sasthamcotta, (d) Point Calimere and (e) Vembanad. Water quality analysis was performed across the three Kerala wetlands using a systematic sequential approach, beginning with Vembanad wetland for initial model development, followed by Ashtamudi and Sasthamkotta. For Vembanad wetland, Sentinel-2 satellite images, downloaded from the Copernicus site for the time period 2016–2024, were clipped using the wetland’s shapefile. Seven sample locations within Vembanad were chosen for field water quality measurements over different time period. Corresponding satellite images for those periods were selected from the clipped dataset. Table 1 summarises the seven sampling locations with coordinates and descriptions and Fig. 3 visualises Vembanad Lake and the sampling points.

**Figure 4.**
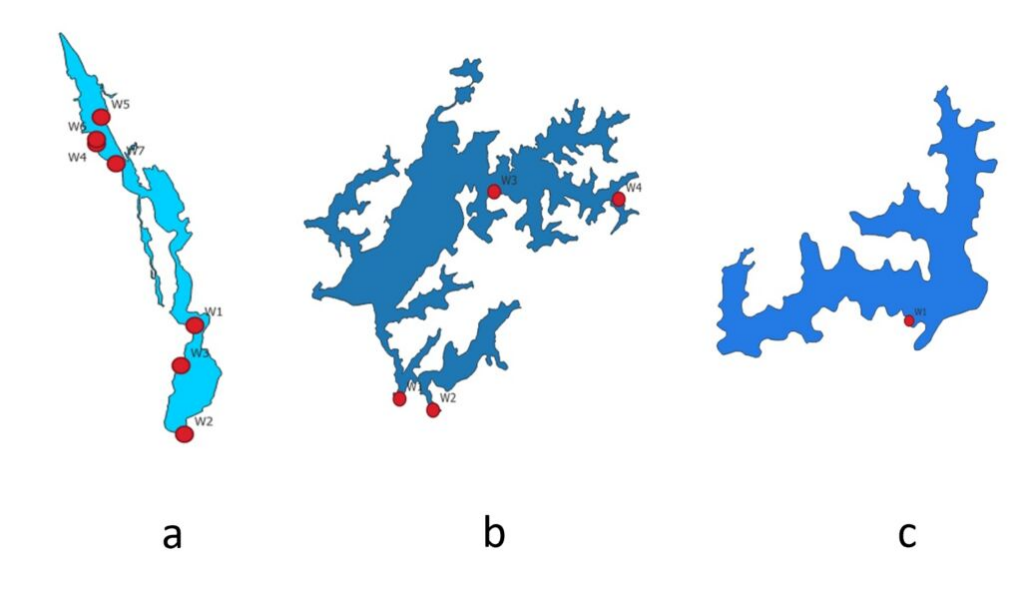
Vembanad field sampling locations.

Sampling point count varied across wetlands due to differences in area, ecological zonation and accessibility. Vembanad Lake, being significantly larger and ecologically complex, had a greater number of sampling points (n = 7) covering spatial gradients, open water zones and anthropogenically influenced areas. Smaller wetlands, such as Ashtamudi and Sasthamkotta Lakes, were adequately represented with four points given their comparatively limited spatial extent. The field sampling points used for model calibration and validation are presented in Fig. 4, showing the measurement locations within the boundaries of: (a) Ashtamudi, (b) Vembanad and (c) Sasthamkotta wetlands. Table 2 presents the field measurements of water quality parameters for the Vembanad wetland during different time periods.

Water quality measurements were collected at periodic intervals depending on satellite data availability and field constraints, with observations spanning multiple years (2016–2024). At each sampling location, a single in-situ measurement corresponding to the satellite acquisition date was used. Multiple linear regression models were developed by relating spectral reflectance values from Sentinel-2 with in-situ measured water quality parameters (Elhag et al. 2019, Pizani et al. 2020, Ouma et al. 2020).

**Results and Discussion**

The performance of regression models was evaluated using the coefficient of determination (R²), a fundamental statistical measure that quantifies the proportion of variance in the dependent variable (water quality parameters) that is predictable from the independent variables (Sentinel-2 spectral bands). R² values range from 0 to 1, where 0 indicates no predictive capability and 1 represents perfect prediction. This metric is particularly valuable for remote sensing applications, as it provides a standardised measure for comparing different modelling approaches and assessing the reliability of satellite-derived water quality estimations against ground-truth measurements.

**Comparative regression analysis for remote sensing water quality assessment**

**Figure 5.**
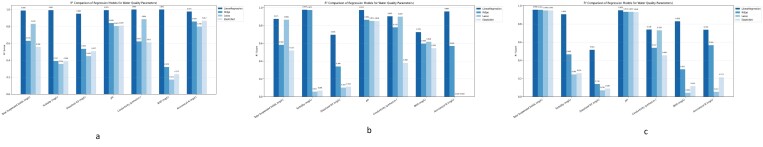
Comparative performance analysis of four regression methods across three study wetlands.

To develop these equations for water quality estimation, four regression modelling approaches were evaluated: Linear Regression, Ridge Regression, Lasso Regression and Elastic Net Regression. All models utilised ten Sentinel-2 spectral bands (B02, B03, B04, B05, B06, B07, B08, B08A, B11, B12) as predictor variables for seven water quality parameters across three study locations. Model performance was assessed using R² scores, with hyperparameter optimisation conducted for regularised models. As illustrated in Fig. 5, the comparative performance analysis for: (a) Ashtamudi, (b) Sasthamcotta and (c) Vembanad, respectively, is presented.

Four regression models (Linear Regression, Ridge, Lasso and ElasticNet) were applied to derive regression equations that relate Sentinel-2 spectral band reflectance values to measured water quality parameters. The primary objective was to identify the best-fit model and extract the corresponding regression equation for predicting water quality parameters from satellite imagery. The R² values reported reflect the goodness of fit between the observed and predicted values. Linear Regression consistently outperformed the other approaches across all study sites and water quality parameters, achieving R² values ranging from 0.725 to 1.000, while regularised models (Ridge, Lasso and Elastic Net) showed significantly lower performance, with many parameters showing R² values below 0.6. The accuracy of Linear Regression, particularly evident in parameters like Turbidity (R² = 0.991), pH (R² = 0.974) and Conductivity (R² = 1.000), led to its adoption for developing the final predictive models for all three study areas. These Multilinear regression models were then applied to all other clipped Sentinel-2 images to estimate water quality parameters over time and space for the study areas.

Multilinear regression models generate quantitative equations that allow remote estimation of these parameters using satellite spectral data alone (Tavora et al. 2023). The regression equations developed for Vembanad wetland water quality estimations are presented in Equations (1) through to (7) in Table 3. Here, B02 to B12 represent the reflectance values of the respective Sentinel-2 spectral bands used in the regression models (Torres-Bejarano et al. 2020). These regression equations enable water quality estimation from all Sentinel-2 images of Vembanad beyond field campaigns, ensuring comprehensive spatiotemporal monitoring. The same methodology was then applied to Ashtamudi wetland. Using its shapefile, satellite images were clipped and sample locations identified for field measurements. Regression models linking satellite reflectance and water quality parameters were similarly developed and applied to the full dataset for Ashtamudi to generate spatially and temporally detailed water quality data. Table 4 summarises the four sampling locations with coordinates and descriptions and the corresponding regression equations for Ashtamudi wetland water quality estimations are expressed in Equations (8) through to (14) in Table 5.

Corresponding regression equations were also developed for Sasthamcotta wetland. For Harike and Point Calimere wetlands, water quality parameter estimations were performed using the Se2WaQ script, a publicly available empirical modelling tool implemented on the Sentinel Hub platform. By applying the Se2WaQ script to the clipped raster datasets for each wetland, we generated consistent and spatially detailed estimations of surface water quality for Harike and Point Calimere (sentinel hub). Following water quality estimation across the five wetlands, bird populations were assessed within the same study areas and time period. This analysis focused on resident and migratory waterbird species. Bird count data were sourced from eBird and regional ornithological surveys conducted by organisations including the Cochin Natural History Society (CNHS), World Wide Fund for Nature – Kerala (WWF), Kottayam Natural Society and Bird Count India. To address data duplication and uneven sampling effort, a standardisation procedure was applied wherein, for dates with multiple observer records, the maximum reported count was retained and duplicates were removed. Year-wise counts were derived from filtered records. The Vembanad dataset includes counts of both resident and migratory waterbird species. Table 6 provides detailed species-wise descriptions of both migratory and residential waterbird counts from 2016 to 2024. The dataset encompasses a wide range of species, such as the Lesser Whistling-Duck (*Dendrocygna javanica* (Horsfield, 1821)), Garganey (*Spatula querquedula* (Linnaeus, 1758)), Northern Pintail (*Anas acuta* Linnaeus, 1758), Gray-headed Swamphen (*Porphyrio poliocephalus* (Latham, 1801)), Eurasian Coot (*Fulica atra* Linnaeus, 1758), Whiskered Tern (*Chlidonias hybrida* (Pallas, 1811)), Little Cormorant (*Microcarbo niger* (Vieillot, 1817)), Indian Cormorant (*Phalacrocorax fuscicollis* Stephens, 1826) and Black-headed Gull (*Chroicocephalus ridibundus* (Linnaeus, 1766)).

In the case of the Vembanad wetland, the highest overall number of waterbirds was recorded in 2016, with approximately 27,398 individuals, while the lowest count was observed in 2021, with around 3043 birds. Similarly, bird population estimates were conducted for the remaining wetlands. For the Harike wetland, the recorded waterbird species included the Lesser Whistling-Duck (*Dendrocygna javanica* (Horsfield, 1821)), Gray-headed Swamphen (*Porphyrio poliocephalus* (Latham, 1801)), Eurasian Coot (*Fulica atra* Linnaeus, 1758), Little Cormorant (*Microcarbo niger* (Vieillot, 1817)), Indian Cormorant (*Phalacrocorax fuscicollis* Stephens, 1826), Indian Pond-Heron (*Ardeola grayii* (Sykes, 1832)), Little Egret (*Egretta garzetta* (Linnaeus, 1766)), Black-headed Ibis (*Threskiornis melanocephalus* (Latham, 1790)), Garganey (*Spatula querquedula* (Linnaeus, 1758)), Northern Pintail (*Anas acuta* Linnaeus, 1758), Whiskered Tern (*Chlidonias hybrida* (Pallas, 1811)), Black-headed Gull (*Chroicocephalus ridibundus* (Linnaeus, 1766)) and Glossy Ibis (*Plegadis falcinellus* (Linnaeus, 1766)).

For Ashtamudi wetland, the species counted included the Lesser Whistling-Duck (*Dendrocygna javanica* (Horsfield, 1821)), Little Cormorant (*Microcarbo niger* (Vieillot, 1817)), Indian Cormorant (*Phalacrocorax fuscicollis* Stephens, 1826), Indian Pond-Heron (*Ardeola grayii* (Sykes, 1832)), Little Egret (*Egretta garzetta* (Linnaeus, 1766)), Black-headed Ibis (*Threskiornis melanocephalus* (Latham, 1790)), Cotton Pygmy-Goose (*Nettapus coromandelianus* (Gmelin, 1789)), Whiskered Tern (*Chlidonias hybrida* (Pallas, 1811)) and Glossy Ibis (*Plegadis falcinellus* (Linnaeus, 1766)).

In the case of Sasthamcotta wetland, the observed waterbird species were the Lesser Whistling-Duck (*Dendrocygna javanica* (Horsfield, 1821)), Gray-headed Swamphen (*Porphyrio poliocephalus* (Latham, 1801)), Whiskered Tern (*Chlidonias hybrida* (Pallas, 1811)), Little Cormorant (*Microcarbo niger* (Vieillot, 1817)), Black-headed Ibis (*Threskiornis melanocephalus* (Latham, 1790)), Indian Pond-Heron (*Ardeola grayii* (Sykes, 1832)), Little Egret (*Egretta garzetta* (Linnaeus, 1766)), Great Egret (*Ardea alba* Linnaeus, 1758) and Medium Egret (*Ardea intermedia* Wagler, 1829).

These species lists reflect the diversity and ecological significance of each wetland, providing a foundation for further analysis of habitat suitability and temporal trends in waterbird occupancy.

**Challenges in collecting data**

The challenges in collecting data were divided into two types: field measurements of water quality parameters and bird data collection. Initially, several water quality parameters were selected for the study.

Since the study spans multiple years, maintaining data consistency was challenging. Some water quality parameters were missing in certain years, so parameters were reduced to seven to ensure consistency. Matching field data with Sentinel-2 imagery acquired on the same dates was also difficult given the extended data collection period from 2016 onwards.

Birdwatchers visiting the same site multiple times create duplicate records, while varying expertise levels affect accuracy. Inconsistent survey routes and timing make comparisons difficult. Platforms like eBird allow random submissions, causing uneven coverage with Sasthamcotta having data only for 2023–2024. Without systematic monitoring, temporal gaps make tracking long-term population changes challenging, highlighting the need for standardised bird monitoring.

## Geographic coverage

### Description

Five Ramsar wetlands in India, three in Kerala (Vembanad 9°34'59.99" N, 76°24'59.99" E; Ashtamudi 8°58'59.99" N, 76°35'59.99" E; Sasthamcotta 9°01'48.00" N, 76°37'48.00" E), one in Punjab (Harike 31°10'12.00" N, 75°12'0.00"E ) and one in Tamil Nadu (Point Calimere 10°17'16.08" N, 79°51'54.36" E).

### Coordinates

 and Latitude; and Longitude.

## Usage licence

### Usage licence

Other

### IP rights notes

**Creative Commons Attribution 4.0 International**

## Data resources

### Data package title

Integrated Dataset of Satellite-Derived Water Quality Parameters and Bird Populations in Five Ramsar Wetlands of India Using Sentinel‑2 Imagery and in‑Situ Observations

### Resource link

https://doi.org/10.5281/zenodo.17557678

### Number of data sets

2

### Data set 1.

#### Data set name

Satellite-Derived Water Quality and Bird Data from Indian Ramsar Wetlands https://zenodo.org/records/17557678

#### Description

A multi-year dataset of water quality parameters derived from Sentinel-2 imagery was compiled for five Ramsar wetlands in India, three in Kerala Vembanad (9°34'59.99" N, 76°24'59.99" E), Ashtamudi (8°58'59.99" N, 76°35'59.99" E), Sasthamcotta (9°01'48.00" N, 76°37'48.00" E) and one in Tamilnadu Point Calimere (10°17'16.08" N, 79°51'54.36" E) and Punjab Harike (31°10'12.00" N, 75°12'0.00" E), using QGIS-defined boundaries and Copernicus satellite data from 2016–2024 (Kerala) and 2018–2024 (others). Thirteen spectral bands were resampled to 10-m resolution and used with regression models based on in-situ measurements to estimate water quality indicators: TSS, turbidity, conductivity, pH, ammoniacal nitrogen, BOD and DO for Kerala; chlorophyll-a, cyanobacteria, turbidity, CDOM and DOC for Harike and Point Calimere.

**Data set 1.** 

Column label	Column description
Point	Sampling location where the measurement was taken.
decimalLatitude	The latitude coordinate of the sampling point in decimal degrees.
decimalLongitude	The longitude coordinate of the sampling point in decimal degrees.
Date	The date on which the water quality parameter was recorded.
Parameter	The name of the water quality parameter measured.
**Unit**	Unit corresponding to the recorded parameter value.
Value	The measured numerical reading for the specified parameter.

### Data set 2.

#### Data set name

Multi-year Bird Population Dataset from Five Ramsar Wetlands in India

#### Description

Dataset contains bird population data compiled from multiple sources, including eBird, CNHS and Bird Count India, for the same five Ramsar wetlands. Each record represents an observation of a particular bird species at a defined location and year. These data cover both resident and migratory species, three in Kerala Vembanad (9°34'59.99" N, 76°24'59.99" E), Ashtamudi (8°58'59.99" N, 76°35'59.99" E), Sasthamcotta (9°01'48.00" N, 76°37'48.00" E) and one in Tamilnadu Point Calimere (10°17'16.08" N, 79°51'54.36" E) and Punjab Harike (31°10'12.00" N, 75°12'0.00" E).

**Data set 2.** 

Column label	Column description
Point	Sampling location where the bird count was conducted.
decimalLatitude	The latitude coordinate of the site in decimal degrees.
decimalLongitude	The longitude coordinate of the site in decimal degrees.
Year	The year in which the bird observation or count was recorded.
Scientific_Name	The scientific name of the observed bird species, including the author and year of its formal description.
Vernacular_Name	The common name of the bird species.
Migratory_Status	Indicates whether the species is migratory or resident.
Individual_Count	The total number of individuals of that species recorded at the site.

## Figures and Tables

**Table 1.** Location of water samples in Vembanad Lake.

Sl. no	Latitude / Longitude	Sample code	Sampling location
1	9°40'55.61" N, 76°23'39.55" E	W1	Vembanad Lake at the U/S of Thanneermukkam bund
2	9°31'7.39" N, 76°22'47.75" E	W2	Vembanad Lake at Padashekaram at Kainikkari
3	9°37'17.38" N, 76°22'31.91" E	W3	Lake Vembanad at Pathiramanal (Alappuzha)
4	9°57'6.95" N, 76°15'41.00" E	W4	Kochi Lake Near Cochin Port Trust
5	9°59'31.03" N, 76°15'59.76" E	W5	Kochi Lake at Goshree Bridge
6	9°57'29.02" N, 76°15'41.00" E	W6	Vembanad Lake,Kochi (Oil Tanker Jetty)
7	9°55'21.66" N, 76°17'12.88" E	W7	Kochi Lake Near Wellington Island

**Table 2.** Field measurements of seven water quality parameters across different time periods at Vembanad.

Date	Total Suspended Solids (mg/l)	Turbidity (mg/l)	Dissolved O_2_ (mg/l)	pH	Conductivity (µmho/cm)	BOD (mg/l)	Ammonical-N (mg/l)
2024 Jan	3.3	2.2	6.52	6.71	2373.5	1.51	0.4
2024 Feb	11	1.51	5.73	7	6768	2.32	0.4
2024 May	31.12	1.34	5.35	7	9566.33	3	0.4
2024 Dec	10.64	2.07	4.87	7.33	2450.85	2	0.4
2023 Jan	53	1.96	7.34	7.65	18641.5	1.82	0.05
2023 Feb	55	7.32	5.22	7.95	16678.8	2.5	0.4
2023 Oct	10.25	7.4	5.86	8.18	11934	2.225	0.4
2022 Feb	51.5	0.5	5.75	7.5	20326	2.65	0.4
2022 Aug	27.5	8.75	6.35	6.9	277	2.6	0.05
2022 Dec	19.25	2.15	5.05	7.45	8989.5	2.65	0.4
2021 Dec	19.5	1.25	5.75	7.2	14970	1.85	0.4
2020 Jan	26.5	1.35	7.25	7.4	980.5	1.1	0.4
2020 Feb	23.5	1.05	5.75	7.25	21596	2.2	0.4
2019 Jan	17.5	2.8	5.75	7.2	18487	3.15	0.05

**Table 3.** The regression equations developed for Vembanad wetland.

**Dependent variable**	**Equation**	
TSS	25.68 + (64.63*B02) - (233.58*B03) + (185.9974*B04) + (67.70*B05) - (195.79*B06) + (1.13*B07) + (99.85*B08) + (189.25*B08A) - (210.75*B11) + (98.35*B12)	(1)
Turbidity	2.9762 - (14.17*B02) - (12.66*B03) + (59.26*B04) - (29.49*B05) + (19.56*B06) - (32.56*B07) + (8.33*B08) +(17.82*B08A) - (19.91*B11) + (10.09*B12)	(2)
Dissolved O_2_	5.8983 + (7.24*B02) - (26.78*B03) + (31.25*B04) - (12.48*B05) - (6.06*B06) - (3.25*B07) - (0.87*B08) + (19.30*B08A) - (10.97*B11) + (7.40*B12)	(3)
pH	7.33 + (1.92*B02) - (2.57*B03) + (3.55*B04) - (2.94*B05) - (0.10*B06) - (0.67*B07) + (0.51*B08) - (0.21*B08A) + (0.83*B11) + (0.14*B12)	(4)
Conductivity	11002.78 + (3692.01*B02) + (102894.41*B03) - (185469.60*B04) + (90265.77*B05) - (9115.64*B06) + (48672.08*B07) - (5251.32*B08) - (100908.42*B08A) + (75411.11*B11) - (46048.87*B12)	(5)
BOD	2.25 - (6.81*B02) +( 23.14*B03) - (27.38*B04) + (11.15*B05) + (5.61*B06) + (5.14*B07) + (2.29*B08) - (21.18*B08A) +(10.86*B11) - (8.16*B12)	(6)
NH3-N	0.3250 - (0.08*B02) - (1.19*B03) + (1.38*B04) + (0.79*B05) - (1.76*B06) - (0.06*B07) + (0.25*B08) + (2.33*B08A) - (1.99*B11) + (1.05*B12)	(7)

**Table 4.** Location of water samples in Ashtamudi Lake.

Sl no	Latitude / Longitude	Sample code	Sampling location
1	8°58'59.99" N, 76°35'59.99" E	W1	Ashtamudi Lake at thoppilakadavu, kollam
2	8°53'28" N, 76°35'6"E	W2	Ashtamudi Lake near ksrtc bus depot, kollam
3	8°58'15.75" N, 76°36'35.60" E	W3	Ashtamudi Lake at perumon, kollam
4	8°58'6.31" N, 76°39'34.66" E	W4	Ashtamudi Lake near m/s. kundara ceramics

**Table 5.** Regression equations for Ashtamudi wetland.

**Dependent variable**	**Equation**	
TSS	1491 + (47756.10*B02) + (59790.59*B03) - (305175.66*B04) + (292806.81*B05) - (170431.49*B06) + (205394.33*B07) + (40813.21*B08) - (209945.51*B08A) + (182511.94*B11) - (142901.96*B12)	(8)
Turbidity	2.7667 - (72.42*B02) - (27.72*B03) + (95.64*B04) - (85.38*B05) + (100.60*B06) - (10.69*B07) + (9.53*B08) + (29.20*B08A) - (139.87*B11) + (101.12*B12)	(9)
Dissolved O_2_	3.9375 + (49.42*B02) - (11.25*B03) + (54.78*B04) - (65.81*B05) - (53.91*B06) - (115.18*B07) - (27.02*B08) + (150.02*B08A) + (27.53*B11) - (8.29*B12)	(10)
pH	7.3542 - (6.25*B02) + (15.25*B03) - (34.63*B04) + (28.16*B05) - (0.29*B06) + (44.89*B07) + (7.96*B08) - (55.11*B08A) + (4.47*B11) - (4.80*B12)	(11)
Conductivity	26672.0833 - (215750.47*B02) + (234770.16*B03) - (712565.02*B04) + (672935.76*B05) + (246554.22*B06) + (906231.27*B07) + (191097.58*B08) - (1273458.78*B08A) + (68965.62*B11) - (124600.59*B12)	(12)
BOD	6.8937 - (104.61*B02) + (149.64*B03) -(272.60*B04) + (244.87*B05) + (63.89*B06) + (310.04*B07) + (78.61*B08) - (406.94*B08A) - (112.17*B11) + (48.36*B12)	(13)
NH3-N	0.3042 + (5.49*B02) - (0.67*B03) - (0.40*B04) + (1.14*B05) - (9.21*B06) + (5.86*B07) - (1.35*B08) - (3.93*B08A) + (7.55*B11) - (4.44*B12)	(14)

**Table 6.** Species-wise waterbird counts in Vembanad wetland (2016–2024).

Residential water birds							Migratory water birds				
Year	*Dendrocygna javanica*	*Porphyrio poliocephalus*	*Fulica atra*	*Microcarbo niger*	*Phalacrocorax fuscicollis*	*Ardeola grayii*	*Egretta garzetta*	*Threskiornis melanocephalus*	*Plegadis falcinellus*	*Chroicocephalus ridibundus*	*Chlidonias hybrida*	*Anas acuta*
2024	4247	301	84	2638	315	728	212	244	854	40	1915	1
2023	1071	129	3	4851	839	1133	122	411	3725	0	2072	408
2022	484	144	9	3607	426	627	83	434	1399	32	1077	0
2021	1638	4	0	326	66	182	74	82	43	0	102	0
2020	1193	317	0	2924	265	254	73	51	3	34	1041	0
2019	1332	1081	21	2646	519	679	322	77	23	394	2176	752
2018	12464	919	110	3320	656	762	153	853	678	80	1094	2624
2017	5592	188	34	2727	500	339	170	675	387	80	795	1522
2016	25953	259	1	1172	504	636	155	226	402	389	770	16787
